# Digital detection of MCI: single‐handed smartphone screening at‐scale

**DOI:** 10.1002/alz70856_106559

**Published:** 2026-01-09

**Authors:** P. Monroe Butler, Jenny Yang, Matt Hobbs, Philippe Syz, Natalia Silveira, Matt Bianchi, Hanson Lenyoun, Hung Pham, Audrey Gabelle, Marty Sliwinski, Mithun Patel

**Affiliations:** ^1^ Apple Health, Apple Inc, Cupertino, CA, USA; ^2^ Brigham & Women's Hospital, Harvard Medical School, Boston, MA, USA; ^3^ Montpellier Excellence University, Montpellier, France; ^4^ Penn State University, University Park, Penn, PA, USA

## Abstract

**Background:**

The widespread availability of consumer‐grade mobile devices offers novel opportunities to capture everyday cognition. Given the increasing prevalence and delayed diagnosis of cognitive impairment, scalable cognitive health screening is critically needed. Remote, unsupervised cognitive assessments using smart devices have demonstrated feasibility, reliability, and validity. These assessments enable both baseline and repeated cognitive performance measures, incorporating performance averaging and variability analysis to enhance signal detection. This study examines the utility of remote, repeated digital cognitive assessments for mild cognitive impairment (MCI) classification using data from a large virtual observational study.

**Method:**

The Intuition study (NCT05058950) was a remote, observational study enrolling over 23,000 U.S. aging adults who contributed 24 months of longitudinal multimodal data via iPhone and Apple Watch. A custom research application collected routine device usage data, self‐reported health information, and cognitive assessments. The key objectives of this work were to evaluate the feasibility of brief, smartphone‐based cognitive assessments for MCI classification compared to a longer, 30‐minute battery administered on personal computing devices (e.g., laptop/iPad). Additionally, we examined whether cognitive fluctuations observed over monthly time scales could be recapitulated over brief diurnal timescales using high‐frequency ecological momentary assessments. MCI classification was conducted using regression and decision‐tree modeling approaches.

**Result:**

We analyzed data from 16,234 aging individuals (mean age = 65.7 years, SD = 6.9) with and without cognitive complaints and 556 participants diagnosed with MCI (mean age = 67.1 years, SD = 8.3). Baseline MCI classification accuracy was comparable between the brief smartphone screener and the longer laptop/iPad battery (AUROC = 0.72, 95% CI = 0.04). Repeated measures over brief diurnal timescales, incorporating performance averaging and variability, improved MCI classification accuracy similarly to repeated monthly assessments using the longer battery (AUROC = 0.80, 95% CI = 0.02).

**Conclusion:**

This study provides proof‐of‐concept findings for MCI classification using remote, interactive, unsupervised cognitive assessments via smartphones and personal computing devices. Our results support the validity of brief cognitive screeners for remote MCI detection and highlight their potential for tracking at‐risk cognitive trajectories in demographically diverse aging populations.

